# Betulinic Acid-Loaded Oleogel as a Novel Pharmaceutical Formulation for Potential Cutaneous Applications: Development, Characterization, and Biosafety Profile

**DOI:** 10.3390/life15060954

**Published:** 2025-06-13

**Authors:** Andreea Smeu, Daliana Minda, Casiana Boru, Lavinia Vlaia, Vicențiu Vlaia, Cristina Adriana Dehelean, Sergio Liga, George Puenea, Daniela Lucia Muntean

**Affiliations:** 1 Research Centre for Pharmaco-Toxicological Evaluation, Faculty of Pharmacy, “Victor Babes” University of Medicine and Pharmacy, 2nd Eftimie Murgu Square, 300041 Timisoara, Romania; 2 Discipline of Toxicology, Drug Industry, Management and Legislation, Faculty of Pharmacy, “Victor Babeş” University of Medicine and Pharmacy Timisoara, 2nd Eftimie Murgu Square, 300041 Timisoara, Romania; 3 Department of Pharmacognosy, Faculty of Pharmacy, “Victor Babeș” University of Medicine and Pharmacy, Eftimie Murgu Square, No. 2, 300041 Timisoara, Romania; 4 Department of Medicine, Vasile Goldis Western University of Arad, Street Liviu Rebreanu, No. 86, 310048 Arad, Romania; 5 Department of Pharmaceutical Technology, Faculty of Pharmacy, “Victor Babeş” University of Medicine and Pharmacy, Eftimie Murgu Square 1, 300041 Timişoara, Romania; 6 Department of Organic Chemistry, Faculty of Pharmacy, “Victor Babes” University of Medicine and Pharmacy, 2nd Eftimie Murgu Square, 300041 Timisoara, Romania; 7 Department of Applied Chemistry and Engineering of Organic and Natural Compounds, Faculty of Chemical Engineering, Biotechnologies and Environmental Protection, Politehnica University Timisoara, Vasile Pârvan No. 6, 300223 Timisoara, Romania; 8 Department of Rehabilitation, Physical Medicine and Rheumatology, “Victor Babes” University of Medicine and Pharmacy, 300041 Timisoara, Romania; 9 Faculty of Pharmacy, “George Emil Palade” University of Medicine, Pharmacy, Science and Technology of Târgu Mureș, 540142 Târgu Mureș, Romania

**Keywords:** oleogel, betulinic acid, novel formulation, textural analysis, FTIR, biosafety, irritant effect, 2D/3D skin models, HET-CAM

## Abstract

Skin disorders constitute a persistent health problem, covering both acute and chronic conditions that manifest in patients of all ages. Betulinic acid (BA) is a triterpene previously studied as an efficient treatment of skin ailments due to its innate pharmacological properties. Nonetheless, due to its lipophilic nature and low bioavailability, topical delivery systems are necessary for its proper administration. Oleogels are efficient carriers for the incorporation of hydrophobic biomolecules; however, their use for the delivery of BA remains scarce. Therefore, this study was designed to develop, characterize, and evaluate a BA-containing oleogel (BA-O) regarding its cutaneous safety profile as a potential pharmaceutical formulation targeting dermatologic issues. The findings illustrated the efficient formulation of BA as oleogel, the product presenting the specific conditions of topical semi-solid formulations in terms of physico-chemical characteristics and high biocompatibility in vitro and in ovo, as BA-O lacked a cytotoxic effect in HaCaT and JB6 Cl 41-5a skin cells (cell viability percentages being > 70%) and was categorized as non-irritant in EpiDerm™ tissues (viability > 80%) and on the chorioallantoic membrane (Irritation Score = 0.186). These results present the preclinical biosafety profile of BA-O with prospective potential for cutaneous applications that should be investigated in future studies.

## 1. Introduction

Skin represents the largest organ of the human body and a part of the integumentary system, being composed of the epidermis, dermis, and hypodermis [1]. Skin diseases are a public health burden affecting between 30% and 70% of the global population, with over 300 acute and chronic conditions described that can occur in patients of all ages. Some of the conditions can be fatal, while others are chronic and can affect patients’ quality of life, such as psoriasis or atopic dermatitis [2]. Skin sensitivity is an important aspect; in fact, epidemiological studies have shown that patients with cutaneous disorders such as rosacea, acne, psoriasis, and atopic dermatitis suffer from a much higher skin sensitivity [3]. Prolonged exposure to hazardous formulations, agents, or substances can cause a plethora of problems that compromise patients’ health, ranging from cutaneous allergies and skin sensitivity to more serious conditions, such as cancer or reproductive problems. Additionally, many compounds used in cosmetics have been categorized as substances with carcinogenic potential, among the most recognized being chemicals [4]. Skin is an organ that plays manifold functions and hosts immunologic and inflammatory processes. Hence, the integrity of the skin barrier is essential to ensure the proper execution of all roles, whereas an intact barrier implies a physiological keratinization process and a normal cutaneous microbial flora [5]. Cell death can cause inflammatory events, and thereby, excess or regular cell death can lead to chronic inflammatory processes. In addition, the epithelial membrane orchestrates multifaceted protection for the cutaneous organ. Cutaneous destruction can occur at the epidermal, dermal, and hypodermal levels, each of which has a well-defined function. The epidermis is the most superficial component, being a stratified keratinized squamous epithelium, with keratinocytes representing the principal structural elements that play an immunomodulatory role [6].

Medicinal plants and compounds of botanical origin used in medical sciences have been recognized since ancient times and by different cultures [7] as being excellent reservoirs of pharmacological properties through the compounds they contain, with triterpenes constituting one of the examples, found in vast and intensively explored sources for their potential biological activities, which have boosted interest in the scientific field [8]. Betulinic acid (BA) is a pentacyclic triterpene obtainable through various techniques, including birch extraction, chemical synthesis, or microbial biotransformation [7]. BA exhibits multiple therapeutic properties, including anti-inflammatory, antibacterial, antimalarial, anticancer, antioxidant, and anti-HIV [9]. Also, BA has already demonstrated its potential use in cosmetics or in the treatment of different skin diseases (e.g., psoriasis, and human melanoma) [10,11,12,13]. In particular, BA alleviates psoriatic and inflammatory skin symptoms, decreases the levels of proinflammatory cytokines in skin lesions (e.g., IL-17A, IL-6), inhibits NF-κB signaling, stimulates collagen, and exhibits antitumor effects observed in cutaneous melanoma cell lines [11,12,13,14]. However, even though it holds numerous benefits for cutaneous applications, BA is instilled with several disadvantages, such as high lipophilicity and low water solubility [15,16]; these limitations underscore the emergent need for the development of new systems for its proper delivery. As regards the cutaneous application of BA, preceding studies have favored its incorporation in gel-based formulations. One such example is the work of Wang et al., who developed a nanostructured lipid carrier (NLC)-based gel containing BA that presented an augmented drug penetration and retention into the skin [17]. Another study focused on the integration of BA into a nanogel vehicle, which demonstrated higher inhibition of skin inflammation in vivo compared to free BA [18]. One promising tool for the delivery of hydrophobic compounds with bioactive activities is oleogel-based formulation [19], which is gaining growing attention for pharmaceutical applications, presenting qualities such as stability, smoothness, and controlled release ability [20]. A study on 45 patients assessing a betulin-based oleogel (prepared from a triterpene extract) suggested that the formulation represents a new approach to the treatment of actinic keratoses, possessing anti-inflammatory and antitumoral effects and being well-tolerated in patients [21]. Dédée F Murrell et al. evaluated the safety and efficacy profile of a birch bark extract oleogel (which included betulin, BA, lupeol, oleanolic acid) in patients with epidermolysis bullosa over a 24-month period, where results showed high tolerability and a reduction in skin lesions upon long-term administration of the oleogel [22]. A group of researchers has explored a new concept approach to develop an oleogel based on birch outer bark extract (containing, among others, BA and betulin) stabilized on extract particles, leading to a more structured network formation and a good tolerance to time-dependent deformation [23]. Also, the development of oleogels with betulin and lupeol showed biocompatibility on HaCaT cells and the ability to exert wound-healing effects [24]. Thus, oleogels have been successfully used as vehicles for the topical administration of various pentacyclic triterpene extracts or betulin targeting several skin conditions, while their tinted use in the formulation of products containing BA as an individual active ingredient for cutaneous applications remains currently underexplored.

Therefore, the present study focused on the preparation and characterization of a BA-loaded oleogel with prospective dermatologic applications and the assessment of its biosafety and biocompatibility using 2D and 3D in vitro skin models (immortalized human keratinocytes, epidermal cells, reconstructed human epidermal microtissues) and the in ovo chorioallantoic membrane of hen eggs. To the best of our knowledge, the current work is one of the first descriptions of the preclinical biosafety profile of a BA-based oleogel, the novelty fulfilling a gap in the existing literature and opening new perspectives for the topical use of BA.

## 2. Materials and Methods

### 2.1. Materials

The active ingredient, betulinic acid, was obtained from Sigma-Aldrich (Darmstadt, Germany). Sunflower (seed) oil and olive (fruit) oil were purchased from Azelis Romania SRL (Bucharest, Romania). Glyceryl dibehenate (Compritol 888 ATO) was provided by Gattefossé Pharmaceuticals (Saint-Priest, France) as a gift sample. For 2D in vitro experiments, Dulbecco’s Modified Eagle’s Medium (DMEM-30-2002 ™), Eagle’s Minimum Essential Medium (30-2003 ™), dimethylsulfoxide (DMSO, 4-X™), fetal bovine serum, mixture of penicillin/streptomycin, and trypsin-EDTA solution were procured from American Type Culture Collection (ATCC), Manassas, VA, USA. The phosphate-buffered saline (PBS) and the MTT (3-(4,5-dimethylthiazol2-yl)-2,5-diphenyltetrazolium bromide) viability kit were obtained via Sigma-Aldrich, Merck KGaA (Darmstadt, Germany), while Hoechst 33342 dye and MitoTracker™ Red CMXRos were acquired from ThermoFisher Scientific (Waltham, MA, USA). The EPI-SIT-200 experimental model, the supplements, and the specific media were all purchased from MatTek Corporation, Ashland, MA, USA. The device Cytation 5 (plate reader) and Lionheart FX (automated microscope) were supplied by BioTek Instruments Inc. (Winooski, VT, USA), Olympus IX73 inverted microscope, and the cellSens Dimensions v.1.8. Software from Olympus (Tokyo, Japan) and the SteREO Discovery.V8 stereomicroscope used for the in ovo evaluations were obtained from ZEISS (Jena, Germany).

### 2.2. 2D Cell Culture Conditions

For 2D in vitro studies, the cell lines involved were HaCaT immortalized human keratinocytes (300493; CLS, Eppelheim, Germany) and JB6 Cl 41-5a neonatal BALB/c epidermal cells (CRL-2010™; ATCC, Manassas, VA, USA). Both cell lines were cultivated in its specific culture medium, namely HaCaT cells in DMEM supplemented with a concentration of 10% fetal bovine serum and a 1% antibiotic mixture of penicillin/streptomycin, and JB6 Cl 41-5a epidermal cells in EMEM with 5% fetal bovine serum and also 1% penicillin/streptomycin. The cells were maintained throughout the experiments under standard conditions represented by a 37 °C temperature and 5% CO_2_.

### 2.3. Preparation of Blank and Betulinic Acid Oleogel Formulations

The blank oleogel (vehicle) was prepared by heating at 80 ± 2 °C, 15% *w*/*w* Compritol 888 ATO (glyceryl dibehenate), and 85% *w*/*w* oil phase (a mixture composed of sunflower oil and olive oil in a 2:1 ratio), under stirring at 600 rpm, until a clear and homogeneous mixture was obtained. After that, the hot fluid oily mixture was further stirred at room temperature until it cooled, to obtain the oleogel. To prepare the betulinic acid oleogel, the same procedure was used, except that the active ingredient (0.3% *w*/*w*) was dissolved into the fluid oily mixture of Compritol 888 ATO, sunflower oil, and olive oil, cooled at a temperature of 50 ± 2 °C.

The mixture of vegetable oils corresponds to the requirements set for semi-solid formulations for topical applications, while glyceryl dibehenate is biocompatible, pharmaceutically approved, included in GRAS status, and is also effective in incorporating the active compounds into the matrix formed in the oleogel [24]. Both preparations (blank oleogel and betulinic acid oleogel) were stored in well-closed containers at room temperature and protected from light until evaluation tests.

### 2.4. Physico-Chemical Evaluation of the Experimental Gels

#### 2.4.1. Evaluation of Organoleptic Properties and pH

The organoleptic attributes of the experimental oleogels (color, physical appearance, and homogeneity) were visually evaluated by applying a 2–4 mm layer of the sample to a glass slide in accordance with the official recommendations [25]. To determine the pH values of oleogels, the compendial potentiometric method [26] was conducted by using a calibrated SevenExcellence™S400-KITpH-meter (from Mettler Toledo, Columbus, OH, USA). Briefly, the dispersion obtained by stirring for 15 min 1 g of oleogel with 20 mL purified water was filtered through a paper filter, and the filtrate was analyzed. For each sample, three replicate measurements were performed at 25 °C, and the obtained results were expressed as mean ± SD.

#### 2.4.2. Evaluation of Rheological Properties

The flow behavior and viscosity of the oleogel formulations were assessed by the viscosimetric test using a HAAKE RheoStress 1 rheometer (Thermo Fisher Scientific, Karlsruhe, Germany) equipped with cone and plate geometry (35 mm diameter, 2°cone angle, and a 0.105 mm gap size). The flow and viscosity profiles were generated and evaluated with HAAKE RheoWin software version 4.3 (Thermo Fisher Scientific, Karlsruhe, Germany); the shear stress and viscosity values were plotted versus share rate ramp-up (from 0.05 to 100 1/s for 120 s) and ramp-down (from 100 to 0.05 1/s for 120 s). The consistency of the experimental oleogels was measured by penetrometry, in accordance with the specifications of the pharmacopeia [26], using a penetrometer (PNR 12, Petrolab, Speyer, Germany) equipped with a specific microcone and appropriate container. The penetration degree value (in mm), as a consistency-related parameter, was registered and analyzed: high values of this parameter indicate a low consistency. Further, the other component related to consistency, namely the spreadability, was measured by parallel-plate procedure, using the del Pozo Ojeda-SuñéArbussá extensometer, based on the procedures described in the literature [27]. All rheological measurements were performed at 25 ± 2 °C, in triplicate, and the results obtained were presented as mean ± SD.

#### 2.4.3. Evaluation of Texture Analysis

Several textural attributes of the two oleogel formulations were investigated with the help of a texture analyzer (TA.XT Plus, Stable Micro Systems, London, UK), provided with a load cell of 5 kg. Two important tests were carried out, namely the back extrusion test and the spreadability test. All experiments were run at room temperature, analyzing four replicates of each sample, and the results are presented as mean ± standard deviation (SD). Data collection and analysis were performed using the Exponent software version 6.1.18.0 (Stable Micro Systems, London, UK). The back extrusion test, which is a compression test, requires performing the experiments in compression mode, using a compression disc with a diameter of 35 mm attached to a rod. Therefore, a specific amount (50 g) of oleogel sample was weighed, without air incorporation, in the 100 mL container provided with the apparatus. After detecting the surface of the oleogel sample, the extrusion disc continued to descend (in the sample) at a speed of 2 mm/s to a depth of 15 mm and returned to the surface at the same speed. From the force-time profile, several textural parameters were calculated [28]: firmness (in g), consistency (in g∙s), cohesiveness (in g), and viscosity index (in g∙s). The spreadability test, using a specific accessory (TTC Spreadability Rig HDP/SR, Stable Micro Systems, London, UK), was conducted by the following procedure: the oleogel sample was placed in the female cone, avoiding air incorporation, and the male-type conical probe descended with a speed of 3 mm/s pressing out the oleogel between the surfaces of the two cones. The instrument software permitted the measurement of two important parameters: firmness (in g) and spreadability (in g∙s), shown in the specific profile of force values versus time.

#### 2.4.4. Fourier-Transform Infrared Spectroscopy (FTIR) Technique Investigation

FTIR spectra of the betulinic acid (BA)-loaded oleogel and pure betulinic acid were acquired in the spectral range of 3500–500 cm^−1^ using potassium bromide (KBr) pellet techniques under reduced pressure. All spectral measurements were conducted at ambient temperature (25 °C) utilizing a Jasco FTIR spectrophotometer (Jasco, Tokyo, Japan).

### 2.5. Computational Predictions of Betulinic Acid Drug Likeness and Toxic Risk

At first, for the computational characterization of the active agent, betulinic acid, concerning its potential drug-like nature and toxicity, the open-source OSIRIS Property Explorer was used, as mentioned by Dehelean and the group of co-workers [29]. For this objective, the canonical SMILES (simplified molecular-input line-entry system) for betulinic acid was sourced using PubChem [30].

### 2.6. Cell Viability Assessment Using MTT Method

To investigate the influence of the samples in vitro, the cell viability was analyzed via the MTT (3-(4,5-dimethylthiazol-2-yl)-2,5-diphenyltetrazolium bromide) colorimetric technique. Following the 24-h treatment period (with Blank-O and BA-O), 100 µL of fresh medium and 10 µL of MTT were added to each well, and the plate was placed in the incubator. Next, after 3 h of incubation, 100 µL of MTT solubilizing solution was added, and the plate was kept for 30 min at room temperature. In the end, the absorbance was measured at 570 and 630 nm wavelengths by the Cytation 5 device.

### 2.7. Bright-Field Cell Morphology Analysis

To analyze the effect of the test formulations on the morphology of HaCaT and JB6 Cl 41-5a cells, they were cultured in 12-well plates at a density of 1 × 10^5^ cells/well, and treated with Blank-O and BA-O (100–500 µg/mL) for 24 h. After treatment, the cells were photographed and analyzed using an Olympus IX73 microscope and the cellSens Dimensions v.1.8. Software.

### 2.8. Hoechst 33342 Nuclear Staining

Hoechst 33342 staining was conducted to detect the modifications caused by Blank-O and BA-O in the nuclei of HaCaT and JB6 Cl 41-5a cell lines. In short, after 24 h of treatment on the cells, the Hoechst 33342 dye solution was prepared by 1:2000 dilution in PBS, the culture medium was removed from the well plates, the dye solution was added, and the plates were incubated in a dark place and at room temperature for 5–10 min. Subsequently, the staining solution was removed, and the cells were washed three times using PBS. Image processing and analysis were completed using the Lionheart FX microscope and Gen5™ Microplate Data Collection and Analysis Software in version 3.14.

Moreover, using the following formula, the apoptotic index (AI) was determined as follows:

$$AI(\%)=\frac{Number\;of\;apoptotic\;cells}{Total\;number\;of\;cells}$$

### 2.9. Mitochondrial Immunofluorescence Staining Using MitoTracker™ Red CMXRos

The mitochondrial aspect of the cells was evaluated using the immunofluorescence staining MitoTracker™ Red CMXRos. For this examination, the cells were grown in 12-well plates, and when the desired confluence was reached, they were stimulated with Blank-O and BA-O 500 µg/mL. MitoTracker™ Red CMXRos was dissolved in DMSO to a 1 mM target concentration and diluted in the specific culture media to a working concentration of 300 nM, according to the protocol from the manufacturer. Live HaCaT and JB6 Cl 41-5a cells were incubated at 37 °C and 5% CO_2_ with the prepared solution for approximately 30–45 min and then rinsed with PBS (3 times 1000 µL/well). Image processing and analysis were completed using Lionheart FX automated microscope (at magnification 20x) and Gen5™ Microplate Data Collection and Analysis Software in version 3.14.

### 2.10. Skin Irritation Assay—Reconstructed Human 3D EpiDermal Model (EPI-200-SIT)

The EpiDerm^TM^ model (EPI-200-SIT) was purchased from MatTek Corporation (Ashland, MA, USA). The negative control for EPI-200-SIT was Dulbecco’s phosphate-buffered saline (DPBS), while the positive control (PC) was 5% sodium dodecyl sulfate (SDS). The specific media, solutions, and MTT kit components for EPI-200-SIT were supplied by the manufacturer. The irritant effect of Blank-O and BA-O was evaluated by performing the skin irritation assay using the EpiDerm^TM^ 3D Reconstructed Human Tissues, under the provided protocol [31]. After their receipt, the reconstructed human tissues were rinsed and cleaned of agarose, transferred into new 6-well plates containing a volume of 0.9 mL of specific culture medium in each well, and incubated for 1 h at a temperature of 37 °C. After this, the culture medium was replaced, and all the inserts were incubated for another 24 h. Next, the tissues were treated for 1 h with 30 μL of negative control (DPBS), positive control (SDS 5%), Blank-O, and BA-O applied on the top of the microtissues after the removal of culture media. Following treatment, the inserts were washed with DPBS and incubated for a period of 24 h, transferred into new culture plates (with 0.9 mL of fresh medium/well), and incubated for another 18 h. Finally, the inserts were moved to a 24-well plate filled with MTT solution (at a volume of 0.3 mL in each well), incubated for 3 h at 37 °C, transferred to a new 24-well plate containing 2 mL of isopropanol in each well, and shaken for 2 h at room temperature. The absorbance was read at 570 nm using the Cytation 5. The viability was calculated using the formulas presented by Pinzaru et al. [32].

### 2.11. Assessment of the Irritant Potential via HET-CAM Assay

The HET-CAM assay was realized to determine the irritancy potential of BA-O at 500 µg/mL. Preparation of the eggs for this test was conducted as follows: (i) disinfection of the hen eggs with 70% (*v/v*) alcohol and placing them in a horizontal position inside an incubator, specifically designed for this purpose, preheated at 37 degrees Celsius and adequate humidity; (ii) on day four, the eggshell was perforated and 5–7 mL of albumen was collected, and the perforation was covered with adhesive tape; (iii) day five consisted of cutting a window in the superior part of the egg to facilitate the visualization of the vasculature, which was also covered by adhesive tape. The HET-CAM test was applied on the 10th day of incubation, using SLS 1% (sodium lauryl sulfate) as a positive control and H_2_O (ultrapure distilled water) as a negative control to quantify the irritant activity of the samples of interest. Thus, 500 µL of 1% SLS, H_2_O, and BA-O 500 µg/mL were pipetted to the chorioallantoic membrane, and the appearance of signals such as lysis, irritation, or hemorrhage was monitored for a period of 5 min. In order to quantify the irritant potential, photographs were taken at time 0 of sample application (T0) and 5 min/300 s after sample application on the chorioallantoic membrane (T5). The obtained images were realized and analyzed with Discovery v.8 stereomicroscope and using the ZEN core 3.8 software. According to the formula described below, the irritation score (IS) was subsequently determined:

$$IS=5\times \frac{301-H}{300}+7\times \frac{301-L}{300}+9\times \frac{301-C}{300}$$

It represents a measurement parameter that quantifies the irritancy potential of test samples by determining the time when modifications (H-hemorrhage, L-vascular lysis, and C-coagulation) occur at the vascular level. From the results of IS, the test substance can be classified as non-irritant if IS = 0–0.9, irritant if IS = 1–8.9, and severely irritant if IS = 9–21 [33].

### 2.12. Statistical Analysis

Statistical evaluation of experimental datasets was performed with the help of GraphPad Prism software, version 10.2.3 (GraphPad Software, San Diego, CA, USA, www.graphpad.com). The differences between Blank-O and BA-O treated groups and the control were determined using One-way ANOVA and Dunnett’s multiple comparison tests. The statistically meaningful outcomes were scored using “*”: * *p* < 0.05 and **** *p* < 0.0001.

## 3. Results

### 3.1. Organoleptic Properties and pH

**Figure 1:**
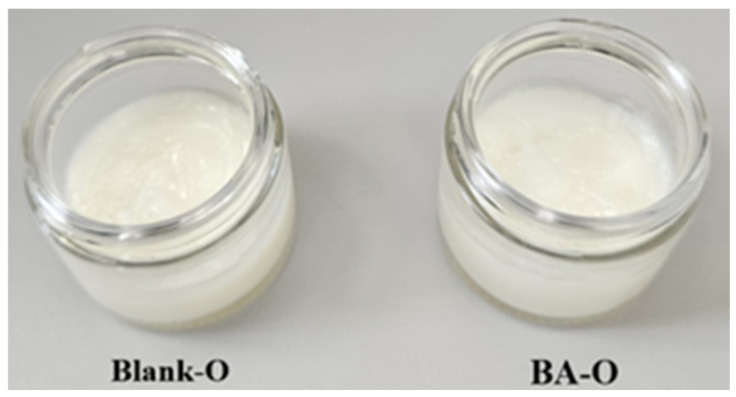
Macroscopic appearance of blank oleogel (Blank-O) and betulinic acid oleogel (BA-O).

The macroscopic properties (appearance, color, odor, and opacity) of the experimental oleogel formulations are presented in Table 1. Also, in Figure 1, the appearance of these preparations is illustrated. From Table 1, it can be noted that the pH values of both experimental oleogels (Blank-O and BA-O) were similar and slightly acidic.

### 3.2. Rheological Properties

**Figure 2:**
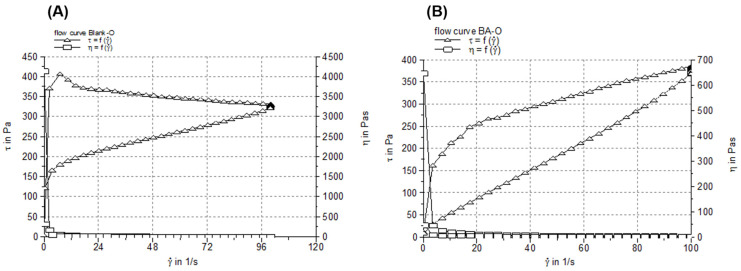
Flow and viscosity profiles of (**A**) blank oleogel (Blank-O) and (**B**) betulinic acid oleogel (BA-O).

The flow and viscosity curves obtained after performing the viscosimetric test are shown in Figure 2, and the viscosity and thixotropy values are listed in Table 2. The flow profiles (Figure 2) indicated that both oleogels behave as non-Newtonian, pseudoplastic (shear-thinning) materials, as the shear stress increased progressively with the applied shear rate. In addition, the flow profiles revealed the thixotropic behavior, correlated with the presence of a hysteresis loop (the region between the shear rate up curve and shear rate down curve). The steady-state viscosity curves of the two samples followed a typical profile of a shear-thinning system. Both oleogels presented very similar viscosity and thixotropy values (Table 2), which suggests that by the incorporation of BA in the oleogel base, no changes in the microstructure of the semisolid vehicle occurred.

**Figure 3:**
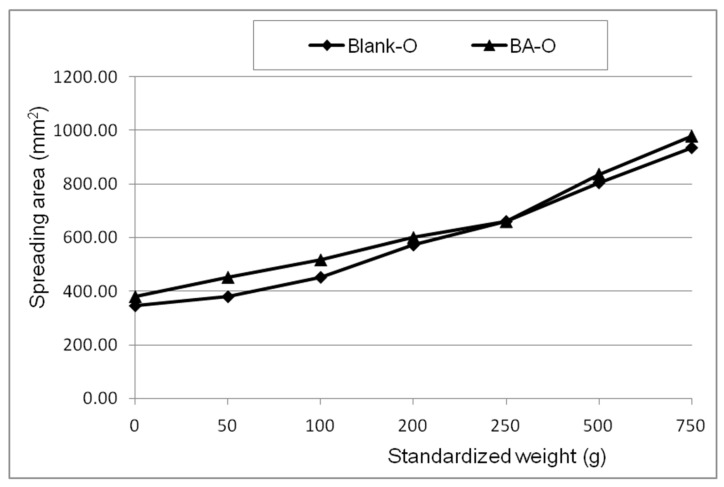
Spreadability profiles of blank oleogel (Blank-O) and betulinic acid oleogel (BA-O).

The spreadability profiles of Blank-O and BA-O formulations, obtained by plotting the surface areas (mm^2^) as a function of applied mass (g), were almost superimposable (Figure 3). The spreadability values of both oleogels indicated that they are smooth, easy-to-spread systems and that they correlate well with the penetration depth values.

### 3.3. Texture Analysis

**Figure 4:**
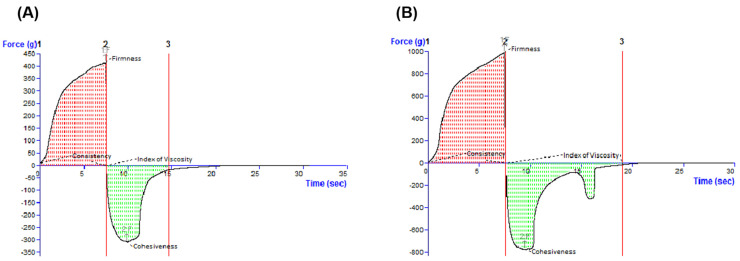
Typical force-time profiles and textural parameters produced in the back extrusion test by (**A**) blank oleogel (Blank-O) and (**B**) betulinic acid oleogel (BA-O).

Analyzing the experimental oleogels by the back extrusion test, typical force as a function of time profiles was obtained (Figure 4), and four specific textural indices were calculated: firmness and consistency (from the positive region of the graph), which reveal the ease of spreading the sample on the skin, and cohesiveness and index of viscosity (from the negative region of the graph), which indicate the sample’s restructuration potential after application and the stickiness of the sample, respectively.

Table 3 presents the values of parameters specific to the back extrusion test. Comparing the Blank-O formulation with that containing betulinic acid in terms of firmness and consistency, it was observed that the latter was approximately 2.4 fold firmer and more consistent. Similarly, based on the cohesiveness and index of viscosity values, BA-O formulation showed a higher stickiness (3-fold) and great ability to rebuild its structure (2.4 times) after application.

**Figure 5:**
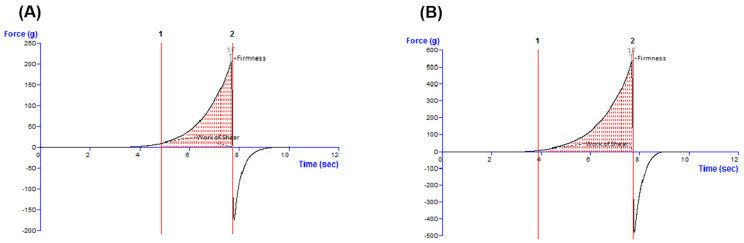
Typical force-time profiles and textural parameters produced in the spreadability test by (**A**) blank oleogel (Blank-O) and (**B**) betulinic acid oleogel (BA-O).

According to Figure 5, the BA-O was 2.6 times firmer and approximately 3 times more spreadable than the Blank-O.

### 3.4. FTIR Analysis

**Figure 6:**
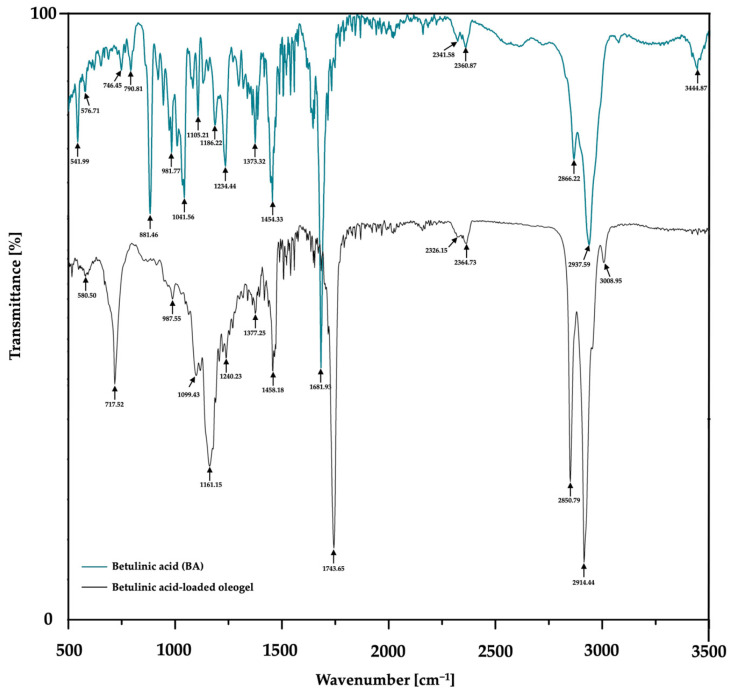
FTIR absorption spectra of betulinic acid (BA) and betulinic acid oleogel (BA-O).

The FTIR analysis was further used for the detection of the functional groups of BA within BA-O in order to demonstrate the incorporation of the active ingredient in the respective formulation (Figure 6).

The FTIR spectrum of pure BA exhibits absorption bands in the high wavenumber region, specifically at 3444.87 cm^−1^, 2937.59 cm^−1^, and 2866.22 cm^−1^. The short band at 3444.87 cm^−1^ is indicative of O–H stretching vibrations, characteristic of intramolecular hydrogen bonding within hydroxyl groups. The peaks at 2937.59 cm^−1^ and 2866.22 cm^−1^ correspond to the asymmetric and symmetric C–H stretching modes of aliphatic CH_3_ and CH_2_ groups, respectively. Additionally, short-intensity bands at 2360.87 cm^−1^ and 2341.58 cm^−1^ are observed, which are typically assigned to weak C–H stretching vibrations. Another strong-intensity band at 1681.93 cm^−1^ is typically attributed to the C=O stretching vibration of the carboxylic acid functional group, which is a key structure of the BA. Within the fingerprint region (500–1500 cm^−1^), the FTIR spectrum of pure BA exhibits several prominent absorption bands, notably at 1454.33, 1373.32, 1186.22, 1041.56, 981.77, 881.46, and 541.99 cm^−1^. These are accompanied by numerous additional bands of medium to weak intensity, reflecting the complex vibrational profile associated with the molecular structure of BA. As also shown in Table 4, the most important absorption peaks recorded for the analyzed oleogel formulation were identified at 2914.44, 2850.79, 2364.73, 2326.15, and 1743.65 cm^−1,^ along with several distinct peaks within the fingerprint region (500–1500 cm^−1^). Even though there are visible absorbance peaks, the essential absorption bands that define BA’s molecular structure are still present in the FTIR spectrum of the BA-loaded oleogel formulation, confirming that its functional groups were retained and incorporated into the oleogel matrix.

### 3.5. Computational Predictions of BA Drug Likeness and Toxic Risk

The computational analysis of the properties retained by BA is furnished in Table 5. The results indicate that BA presents no risk for mutagenic, tumorigenic, irritant, or reproductive toxicity. As regards its drug-like features, although it presents a negative drug-likeness, the overall drug score remains positive.

### 3.6. Cell Viability Evaluation Using the MTT Method

**Figure 7:**
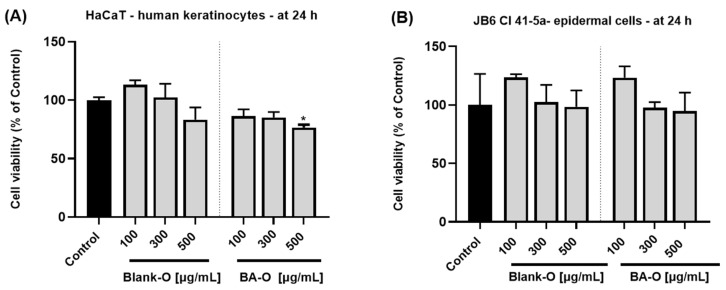
Cell viability percentages 24 h after treatment of HaCaT cells (**A**) and JB6 Cl 41-5a cells (**B**) with blank oleogel (Blank-O) and betulinic acid oleogel (BA-O) (100, 300, and 500 μg/mL). The results are presented as percentages (%) normalized to control (untreated cells). All data are reported as mean values ± SD from three independent experiments done in triplicate. For analyzing the statistical differences between the untreated group (control) and treated groups, the One-way ANOVA test was applied, followed by Dunnett’s multiple comparison post-test. “*” marks statistical significance (* *p* < 0.05).

The initial step in observing the cytotoxic potential of the formulations was the determination of cell viability after a 24 h treatment using the MTT technique. Thus, in the case of treatment in HaCaT cells, as shown in Figure 7A, Blank-O at concentrations of 100 and 300 μg/mL induced a slight cell stimulation, while the concentration of 500 μg/mL reduced the cell viability to the percentage of only 83.31%. HaCaT cells treated with BA-O exhibited a slight dose-dependent reduction in cell viability, with the highest concentration tested of 500 μg/mL achieving a threshold of 76.52%. According to Figure 7B, in JB6 Cl 41-5a cells, Blank-O treatment induced cell stimulation at the lowest concentrations, and the 500 μg/mL concentration led to a cell viability of only 98.49%. For BA-O treatment, the concentration of 100 μg/mL induced cell stimulation up to 123.28%, while the highest concentration of 500 μg/mL reduced cell viability to 94.78%.

### 3.7. Bright-Field Morphological Assessment

**Figure 8:**
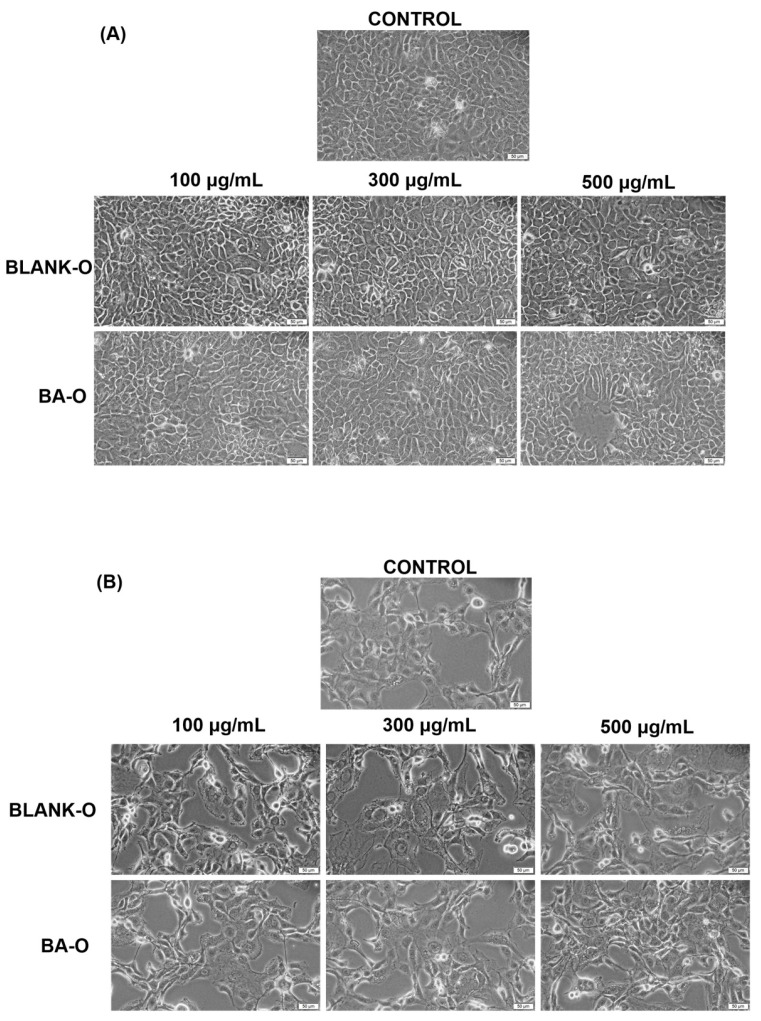
Representative bright-field microscopy images illustrating the morphological changes observed at 24 h of treatment of HaCaT cells (**A**) and JB6 Cl 41-5a cells (**B**) blank oleogel (Blank-O) and betulinic acid-based oleogel (BA-O) (100, 300, and 500 μg/mL). The images were captured at a magnification of 20×, and the scale bar indicates 50 μm.

The next step in performing the biosafety study of the formulations was to analyze the morphology of the cells 24 h after treatment. In HaCaT keratinocyte cells, as illustrated in Figure 8A, treatment for 24 h with Blank-O (100–500 µg/mL) did not induce cell dysmorphologies; the appearance of treated cells, even at the highest concentration of 500 µg/mL, was similar to control (untreated) cells. Likewise, BA-O did not induce changes in cell morphology at any of the concentrations tested (100–500 µg/mL). In JB6 Cl 41-5a epidermal cells (Figure 8B), neither Blank-O nor BA-O (100–500 µg/mL) induced cell dysmorphologies, with cell shape and appearance remaining the same as in untreated cells.

### 3.8. Nuclear Immunofluorescence Staining via Hoechst 33342

**Figure 9:**
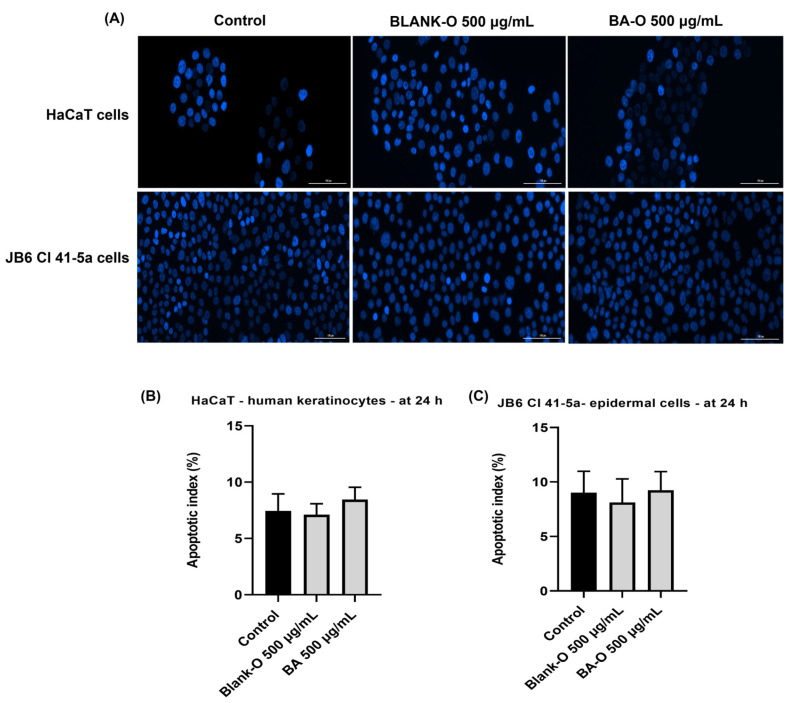
Visualization of nuclear morphology changes at 24 h of treatment of HaCaT cells and JB6 Cl 41-5a cells (**A**) and the apoptotic index (%) for HaCaT (**B**) and JB6 Cl 41-5a (**C**) cells treated with blank-oleogel (Blank-O) and betulinic acid-based oleogel (BA-O) (500 μg/mL). The images were captured at a magnification of 20×, and the scale bar indicates 100 μm.

The next step was to analyze the test samples regarding their nuclear impact. For this assay, the highest concentration investigated, 500 μg/mL, was chosen. The impact on cell nuclei was also assessed for Blank-O and BA-O (500 μg/mL) samples in HaCaT and JB6 Cl 41-5a cells (Figure 9A). Blank-O and BA-O did not induce major nuclear dysmorphologies in HaCaT cells compared to untreated nuclei, the ratio of condensed nuclei being approximately the same in both control and treated cells. In JB6 Cl 41-5a cells, slight changes can be observed, indicated by condensation of nuclei or shrinking in shape, however, in a reduced manner. For both HaCaT and JB6 Cl 41-5a cell nuclei, the confluence of nuclei was unchanged compared to the control. Based on the calculated AI on HaCaT (Figure 9B) and JB6 Cl 41-5a (Figure 9C) cells, Blank-O and BA-O showed no statistically significant changes relative to the control, with similar percentages of apoptotic nuclei.

### 3.9. Mitochondrial Immunofluorescence Staining

**Figure 10:**
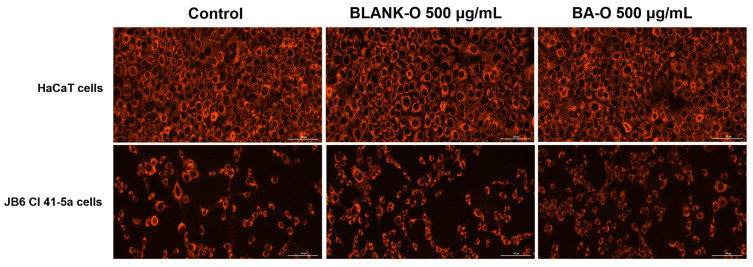
Visualization of mitochondrial morphology changes at 24 h of treatment of HaCaT cells and JB6 Cl 41-5a cells treated with blank oleogel (Blank-O) and betulinic acid-based oleogel (BA-O) (500 μg/mL). The images were captured at a magnification of 20×, and the scale bar indicates 100 μm.

As indicated in Figure 10, HaCaT cells did not undergo mitochondrial alterations after 24 h treatment with Blank-O and BA-O. Also, JB6 Cl 41-5a cells treated with Blank-O and BA-O do not display mitochondrial aberrations, retaining their shape similar to the mitochondria of the control cells.

### 3.10. 3D EpiDerm Skin Irritation Test

**Figure 11:**
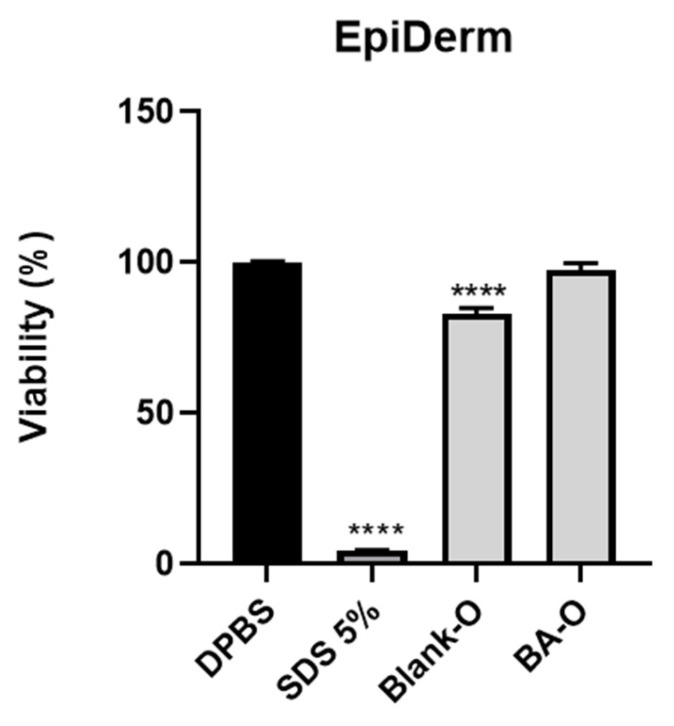
Graphical representation showing the viability percentage of inserts of the EpiDerm skin model (EPI-200 SIT) at 18 h post-treatment with blank oleogel (Blank-O) and betulinic acid oleogel (BA-O). Positive control is represented by SDS 5%, and negative control is represented by DPBS. For analyzing the statistical differences between the control group and treated groups, the One-way ANOVA test was applied, followed by Dunnett’s multiple comparison post-test. (**** *p* < 0.0001).

Regarding the impact of Blank-O and BA-O on EpiDerm skin model inserts (EPI-200 SIT), the results illustrated in Figure 11 suggest that treatment of the epidermal microtissues with Blank-O led to a viability of 83.01%, while BA-O application led to a viability of 97.52%. Also, the SDS 5% positive control induced a massive decrease in viability down to around 4.4%.

### 3.11. In Ovo Irritant Potential via HET-CAM Test

**Figure 12:**
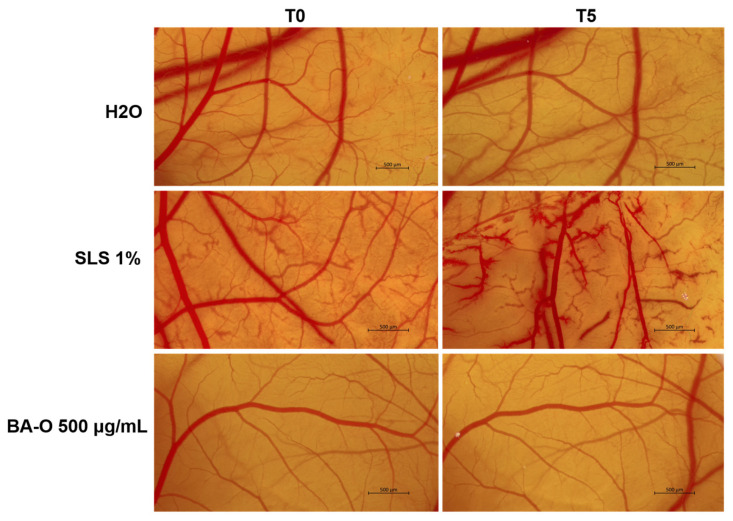
Presentation of the images illustrating the aspect of the chorioallantoic membrane vasculature before treatment (T0) and 5 min after treatment with H_2_O (negative control), SLS 1% (positive control), and betulinic acid-based oleogel (BA-O) 500 μg/mL. The scale bars indicate 500 µm.

The irritant potential of the samples was monitored by the in ovo HET-CAM method, which involves a direct application to the chorioallantoic membrane of the samples of interest. In this way, the irritant effect was examined for BA-O, where SLS 1% was used as a positive control and H_2_O as a negative control. According to the obtained results (Figure 12, Table 6), BA-O is a non-irritant formulation (IS = 0.186), similar to the negative control H_2_O with an IS = 0.069. On the opposite, SLS 1% was classified as a severe irritant agent with IS = 20.37.

## 4. Discussion

Pentacyclic triterpenes are botanical compounds that exert multiple biological effects (i.e., anticancer, anti-inflammatory, antimicrobial), with BA, being one of the family’s representatives as regards the outstanding number of studies dedicated to the exploration of its pharmacological effects, including anti-inflammatory, hepatoprotective, anti-angiogenic, or antitumor [24]. In the dermato-cosmetic field, BA is considered a key constituent because it has been proven to exert anti-aging and collagen-stimulating activities [12,34]. Additionally, specialized studies have confirmed that BA produces anti-aging effects via various targets, one of the mechanisms of action being the regulation of the SITR1-p53 pathway [34]. Nonetheless, the cutaneous application of BA remains limited by its hydrophobic nature [15]. Oleogels became a promising tool for the formulation and delivery of hydrophobic compounds with bioactive profiles [19], although, to date, studies on BA oleogel formulation remain scarce. In light of these aspects, the present research proposed the development, characterization, and biosafety assessment using 2D and 3D in vitro skin models and the vascularized chorioallantoic membrane of fertilized hen eggs of a BA-containing oleogel formulation as a potential dermato-cosmetic product.

The study begins with the characterization of the BA-O novel formulation. According to Figure 1, it can be observed that the pH values of Blank-O and BA-O were similar and under slightly acidic conditions. As these pH values are higher than 4.5 (the limit below which a dermal preparation can produce skin irritation) [35], one can suggest that the oleogel containing BA has good skin tolerance and is suitable for dermal application. For pharmaceutical semisolids, including oleogels, their steady shear flow and viscosity curves, indicating the relationship between the shear stress/viscosity and shear rate, can provide valuable information about their behavior in different circumstances (i.e., when they are dispensed by squeezing the tube, during application on the skin, and their physical stability during shelf life), as various changes in their microstructure can occur [36]. Considering the rheological attributes (Figure 2), high viscosity at low share rates and low viscosity at high share rates, it can be suggested that the two studied oleogels are easy and accurate to apply on the skin and have an extended shelf life, and are easy to spread and rub into the skin (with faster absorption of the active ingredient into the skin). Next, the consistency of oleogel formulations was investigated in terms of hardness (expressed by penetration depth) and spreadability (indicated by the surface areas of the oleogel sample after applying each standardized weight). The hardness of the semisolid samples is inversely proportional to the penetration depth of the cone, while their spreadability is directly proportional to the surface area. As shown in Table 2, Blank-O and BA-O produced similar penetration depth values, which fall within the range of the values provided by the current edition of the European Pharmacopoeia for the consistency of white soft paraffin (60–300 mm) [37], the excipient most frequently recommended for the formulation of ointments. The spreadability of a semisolid reveals the ease of skin application. The spreadability profiles of Blank-O and BA-O, obtained by plotting the surface areas (mm^2^) as a function of applied mass (g), were almost superimposable, as can be observed in Figure 3, and the values of the spreading surfaces fall within the specific range of semisolid dermatological preparations [38]. In addition, the spreadability values of Blank-O and BA-O demonstrated that they are smooth and correlate well with the penetration depth values.

The characterization continued with the textural assessment, which is an important tool providing information about several sensorial attributes, such as extrudability from a container, spreadability on the skin, adhesiveness, and consistency [39]. By texture analysis, these sensorial properties can be quantified as the external force applied to the material [39]. By examining the oleogels by the back extrusion test (Figure 4), it was revealed that the ease of spreading of the sample on the skin. The cohesiveness and the viscosity index (in the negative region of the graph) indicate the restructuring potential of the sample after application and the adhesion of the sample, respectively [40]. Complementarily, a textural test was performed in the back extrusion test and the spreadability test, which is illustrated in Table 3. Consequently, it can be suggested that the incorporation of BA in the oleogel vehicle had a favorable effect on the respective textural properties, as high firmness, consistency, and cohesiveness, and a medium index of viscosity are desired for an optimal topical oleogel formulation [41]. The final step of the rheological analyses was the evaluation of the typical force-time profiles and textural parameters produced in the spreadability test by the Blank-O and BA-O formulations, which are shown in Figure 5. When the spreadability of oleogels was evaluated with a texture analyzer, specific spreadability profiles were registered (Figure 5), and two textural parameters were determined in the positive region of the graphs: the maximum force value and the work of shear corresponding to the area under the curve (Table 3). An FTIR analysis was performed in order to demonstrate the incorporation of the active ingredient, BA, into the composition of the oleogel formulation. Through Figure 6 and Table 4, it was confirmed that the functional groups of BA were retained following oleogel formulation, results that indicate the proper incorporation of this active ingredient.

The second part of the paper focused on the assessment of the biocompatibility and biosafety of the formulations, as these investigations are gold standards for the evaluation of products intended for skin application, considering that therapeutic cosmetics require rigorous safety evaluation according to the Food and Drug Administration, particularly with regard to adverse effects that may occur in the form of skin irritation or allergy [42]. Initially, BA was computationally analyzed in terms of toxic potential and drug-like features using OSIRIS Property Explorer, a very popular and accurate web tool to predict the physicochemical and toxicological profiles of drugs by estimating drug-like properties, mutagenic, tumorigenic, irritant, and reproductive toxicity risks [33]. As shown in Table 5, BA demonstrated no predicted risk for any severe toxic effects and presented a negative drug-likeness but, overall, a positive drug score.

The study continued with in vitro evaluations in terms of the influence of the cytocompatibility of the obtained formulations on skin-derived cells. The epidermis constitutes a stratified squamous epithelium, constantly proliferating, differentiating, and eliminating to maintain the functional integrity of the tissue within parameters [43]. Keratinocytes are the primary functional and structural elements of the epidermis, the most external layer of the skin, being specialized in guarding against external factors and preventing the leakage of body fluids. Alteration of the epidermal barrier and aberrant differentiation of keratinocytes is implicated in the pathophysiology of numerous skin ailments, such as atopic dermatitis [44]. Under these aforementioned aspects, the HaCaT (immortalized human keratinocytes) and JB6 Cl 41-5a (epidermal cells) cell lines were selected for Blank-O and BA-O cutaneous safety assessment. HaCaT cells are very similar to isolated keratinocytes in terms of their morphology and functionality [45]. JB6 Cl 41-5a epidermal cells were selected owing to their epithelial morphology, and since they are commonly used in cutaneous toxicity studies [46]. Furthermore, other studies also opted for the two cell lines to assess cutaneous safety [33]. The chosen concentrations subjected to testing (100–500 μg/mL) were selected based on previous studies evaluating the safety of different gel formulations in similar dose ranges [24,47,48]. As a first step in the biosafety analysis of Blank-O and BA-O, the viability of the cells after a 24-h treatment was determined using the MTT assay. The findings observed in Figure 7A,B demonstrated that treatment with Blank-O did not produce any significant reduction in cell viability. On the contrary, the lowest concentrations used (100 and 300 μg/mL) produced a stimulation in both HaCaT and JB6 Cl 41-5a cells. Upon BA-O treatment, in both HaCaT (Figure 7A) and JB6 Cl 41-5a cells (Figure 7B), the percentages of cell viability remained above 70%; thus, according to ISO 10093–5:2009 standard which states that a substance is cytotoxic only if the cell viability decreases by more than 30% [49], the tested formulations were classified as non-cytotoxic. Comparably, in a study conducted by Pârvănescu et al., oleogel formulations containing betulin and lupeol as active ingredients (62.5–500 μg/mL) showed no sign of cytotoxicity on HaCaT cells at 24 and 72 h of treatment [24]. Also, BA has been individually tested previously in skin cells for safety evaluation. After 24 h treatment of HaCaT cells with BA in the concentration range of 1–50 μM, no signs of cytotoxicity were observed, all percentages being above 80% [14].

The subsequent step was to conduct an evaluation of the morphological appearance of HaCaT and JB6 Cl 41-5a cells after a 24 h treatment with the samples of interest. The results illustrated that, following treatment, there was no evidence of alterations in the appearance of HaCaT (Figure 8A) and JB6 Cl 41-5a (Figure 8B) cells compared to untreated cells for any of the samples tested. In this way, the morphological aspect of Blank-O and BA-O treated cells in the range of 100–500 μg/mL disclosed that there were no signs of cytotoxicity.

Next, the cells were subjected to a post-treatment evaluation of the nuclear aspect. Hoechst 33342 staining is a tool that can help to identify nuclear morphological changes, chromatin condensation, and nuclear fragmentation in early apoptosis [50], thus allowing to observe whether Blank-O and BA-O have the ability to cause damage at the nuclear level. The nucleus is a regulatory center in cells, thus governing multiple aspects of cellular functions (e.g., DNA replication, RNA processing). As a consequence, deviations in nuclear morphology and organization can affect cellular activities [51]. For this aim, the selective DNA staining with the fluorochrome Hoechst 33342 was used, which presents the advantage of being non-toxic [52]. For this type of experiment, the cells were stimulated for 24 h with the highest concentration of Blank-O and BA-O, namely 500 μg/mL. As illustrated in Figure 9, HaCaT cells did not change nuclear shape, and the apoptotic index shown in Figure 9B did not reveal statistically significant changes compared to the control. In JB6 Cl 41-5a cells, Figure 9 depicts that the nuclear shape of treated cells showed no alterations compared to the nuclear shape of untreated cells, and the apoptotic index outlined in Figure 9C emphasized the same trend, showing no statistical significance following treatment.

Mitochondria are organelles that serve an essential function in energy metabolism and control of stress responses, being the principal source of intracellular reactive oxygen species. Given their multiple roles, altered mitochondrial function can lead to a number of harmful consequences that can include metabolic diseases, cancer, and other ailments related to dysregulated cell death [53]. Mitochondria are the main target affected by ultraviolet radiation and chronological aging. Aberrations in mitochondrial DNA are also commonly found in skin lesions, with studies increasingly supporting the association between mitochondria and skin health. Additionally, mitochondria play an indispensable role in the skin, and their dysfunctions also contribute to skin aging. These mitochondrial dysfunctions are accompanied by reduced antioxidant defense mechanisms, increased cell death, and altered cellular respiration [54]. In this regard, mitochondrial damage was also monitored, for which the MitoTracker™ Red CMXRos immunofluorescence staining was applied. The presentation in Figure 10 highlights that HaCaT cells treated with Blank-O and BA-O at 500 μg/mL did not induce any cytotoxic signal, the mitochondrial appearance remaining consistent with control cells. Similarly, the mitochondria of the JB6 Cl 41-5a cells were not affected by the 24 h Blank-O and BA-O 500 μg/mL treatment, with no visible modifications compared to the untreated cells.

The irritant capacity of Blank-O and BA-O was measured in a 3D experimental model. Reconstructed human epidermis (RHE) is also an alternative to animal testing, composed of a structure similar to the human epidermis. Gazel and coworkers demonstrated that human skin and RHE showed overlap in critical markers of epidermal differentiation (including keratinocytes K1, K10, and K2e) [55]. The model used in the evaluations, i.e., EpiDerm, was designed to predict the skin irritation potential according to the European Union classification system, substituting in this manner the acute in vivo skin irritation test in rabbits. The procedure comprises the topical exposure of the test substance to a reconstructed human epidermis (RhE), followed by the viability test, which is measured by the conversion of MTT dehydrogenase to formazan, quantitatively measured after the extraction from the epidermal tissues. The model is formed from normal human-derived epidermal keratinocytes cultured with the intention of producing a highly differentiated multilayered model of the human epidermis [31]. The results illustrated in Figure 11 presented that the treatment with Blank-O and BA-O on the epidermal microtissues led to viability above 50% in both cases, which confirms the lack of irritant potential according to the manufacturer’s specifications [31]. In a similar way, the experimental model EPI-SIT-200 was used by another group of researchers to identify the irritant effects of other formulations for cutaneous application, namely proniosomal gel blank (vehicle) and proniosomal gel loaded with rutin. According to their results, it was identified that both gels produced no irritant effects, the viability after treatment being also above 50% [32].

Lastly, the irritant potential of Blank-O and BA-O was investigated in ovo, by applying the HET-CAM test, a sensitive, rapid, and inexpensive toxicity technique recommended by the Interagency Coordinating Committee for the Validation of Alternative Methods (ICCVAM) for non-regulatory validation or optimization of preclinical studies [56]. The HET-CAM assay is frequently applied in the assessment of irritant effects and is recognized due to its correlation with the results of dermal irritation tests [33]. Following this assay, as reported in Figure 12 and Table 6, BA-O 500 μg/mL was classified as non-irritant with an IS of 0.186, being included in the same category as the negative control used (i.e., H_2_O - IS = 0.069). The vehicle, Blank-O, at the concentration of 500 μg/mL, was previously evaluated in terms of irritant effect by Pârvănescu et al., who determined its IS as being 3.61 [24], which is higher compared to the one obtained for BA-O. In the same study, oleogels containing betulin and lupeol as active ingredients were classified as non-irritants [24], similar to the results obtained for BA-O.

As far as we know, this study constitutes one of the first characterizations of the preclinical safety profile of BA in oleogel form. However, some limitations should be addressed in future investigations. Firstly, in vitro toxicity evaluations of the samples were performed at a 24 h time interval that corresponds to the ISO 10993-5 standard for cytotoxicity assessment, defining an incubation time of 24 h or longer, if necessary, with most laboratories using the minimal incubation time [57]. In this regard, future studies on Blank-O and BA-O investigation should be developed to investigate the safety profile following longer exposure times. Secondly, the current work focused on the evaluation of Blank-O and BA-O using in vitro 2D and 3D skin experimental models and the chorioallantoic membrane vasculature, while future in vivo studies are required to translate the obtained results on their biocompatibility into clinical practice. Future research directions for the study of BA-O should be directed towards the assessment of its potential utilization as a dermato-cosmetic product with anti-aging, anti-inflammatory, wound healing, or anti-tumor effects. Thirdly, throughout the experiments, the evaluated oleogel formulations were maintained under standard conditions (at room temperature, protected from direct light and humidity), presenting no organoleptic modifications. In view of the future translation of BA-O into clinical practice, stability studies over a long period and the related shelf-life verification are fundamental, as the stability of pharmaceuticals constitutes an essential product quality attribute [58], and needs to be included as a future research direction. Fourth, the consistency of BA incorporation into the oleogel was confirmed by the uniform appearance and FTIR analysis of BA-O. However, future examinations should be oriented to the quantification of the BA content in the oleogel using additional methods, and supplementary kinetic studies should be conducted to outline the in vitro release profile of BA from the formulation.

## 5. Conclusions

Overall, the present work concludes that the formulation of BA as an oleogel yields a product that fulfills the specific requirements of topical semi-solid preparations in terms of organoleptic, rheological, textural, and pH properties, while also presenting high biocompatibility both in vitro and in ovo, attributed to its lack of cytotoxicity and irritant potential. The findings presented herein stand as a foundation for future research on the use of BA-containing oleogel for the management of distinct dermatologic conditions.

## Figures and Tables

**Table 1:** Organoleptic parameters and pH values of the experimental oleogels.

Formulation	Organoleptic Characteristics				pH
	Appearance	Color	Odor	Opacity	
Blank-O	Homogeneous, smooth-oily	White-yellowish	Slight odor, specific to vegetable oils	Opaque	6.912 ± 0.08
BA-O	Homogeneous, smooth-oily	White-yellowish	Slight odor, specific to vegetable oils	Opaque	6.918 ± 0.14

**Table 2:** Rheological parameters (steady state viscosity and thixotropy) and penetration depth values of the experimental blank oleogel (Blank-O) and betulinic acid oleogel (BA-O).

Formulation	Blank-O	BA-O
Viscosity (Pa·s)	3.258 ± 0.025	3.765 ± 0.043
Thixotropy (Pa/s)	10,610 ± 103.29	11,290 ± 125.54
Penetration depth (mm)	127.0 ± 1.523	117.7 ± 2.524

**Table 3:** Textural indices values of experimental oleogels produced by textural assays in the back extrusion test and spreadability test (values expressed as mean ± standard deviation, *n* = 3).

Formulation Code	Firmness (g)	Consistency (g·s)	Cohesiveness (g)	Index of Viscosity (g·s)	Work of Shear (g·s)
Back extrusion test					
Blank-O	415.99 ± 4.33	2202.20 ± 14.42	−309.44 ± 3.67	−1226.09 ± 9.37	-
BA-O	991.73 ± 6.28	5095.60 ± 12.43	−780.27 ± 5.76	−3184.24 ± 14.68	-
Spreadability test					
Blank-O	205.33 ± 2.66	-	-	-	206.95 ± 3.04
BA-O	531.98 ± 4.11	-	-	-	602.14 ± 2.57

**Table 4:** FTIR spectra of betulinic acid (BA) and betulinic acid oleogel (BA-O).

Functional Groups	Absorption Bands in Pure Betulinic Acid (cm^−1^)	Absorption Bands in BA-Loaded Oleogel Formulation (cm^−1^)
Aliphatic C–H stretching vibrations	2937.59	2914.44
	2866.22	2850.79
C–H stretching vibrations	2360.87	2364.73
	2341.58	2326.15
C=O stretching vibration of the carboxylic acid functional group	1681.93	1743.65
Fingerprint region (e.g., CH_2_ and CH_3_ groups bending vibrations, multiple C–O stretching vibrations)	1454.33	1458.18
	1373.32	1377.25
	1186.22	1161.15
	1041.56	1099.43
	981.77	987.55
	881.46	717.52
	541.99	580.50

**Table 5:** Computational analysis of the properties exerted by BA using the OSIRIS Property Explorer Program.

Compound	MW	cLogP	Solubility	Drug-Likeness	Drug Score	Mutagenic, Tumorigenic, Irritant, and Reproductive Toxic Potential
BA	456.0	6.37	−6.28	−21.49	0.15	No risk

MW–Molecular weight; cLogP–Partition coefficient.

**Table 6:** Calculated irritation score (IS) for betulinic acid-based oleogel (BA-O) 500 μg/mL using the HET-CAM method. H_2_O was used as the negative control, and SLS 1% represents the positive control.

Sample	IS	Irritation Category
SLS 1%	20.37	Severely irritant
H_2_O	0.069	Non-irritant
BA-O 500 μg/mL	0.186	Non-irritant

## Data Availability

The data presented in this study are available on request from the corresponding author.
